# Editorial: Culture and emotion in educational dynamics, volume V

**DOI:** 10.3389/fpsyg.2026.1837390

**Published:** 2026-04-16

**Authors:** Enrique Riquelme, Darío Páez, Silvia Cristina da Costa Dutra

**Affiliations:** 1 Departamento de Diversidad y Educación Intercultural, Universidad Católica de Temuco, Temuco, Chile; 2 Centro de Investigación, Innovación y Creación, Universidad Católica de Temuco, Temuco, Chile; 3 Universidad Andrés Bello, Santiago, Chile; 4 Departamento de Psicología y Sociología, Universidad de Zaragoza, Zaragoza, Spain

**Keywords:** culture, dynamics, education, emotion, learning

This fifth volume of *Culture and emotion in educational dynamics* continues the interdisciplinary exploration of how emotional processes interact with educational practices, cultural contexts, and institutional environments. Building upon the conceptual and empirical foundations established in previous volumes, the studies included in Volume V expand the field by addressing new dimensions of emotional experience in education, including supervision cultures in graduate training, family and cultural influences on learning, teacher emotional regulation, and emerging technological approaches for understanding emotions in learning environments. Across diverse methodological traditions—ranging from qualitative narrative inquiry to large-scale quantitative modeling and systematic reviews—the articles gathered in this volume collectively illustrate the growing complexity of research on emotions in education. Several studies focus on developing and validating measurement instruments capable of capturing culturally situated emotional experiences, while others examine the emotional dynamics that shape learning engagement, motivation, and professional identity among students and teachers. In addition, the volume highlights the broader sociocultural contexts in which educational emotions emerge, emphasizing how institutional structures, family practices, and cultural values influence emotional experiences in learning environments.

The contributions reflect a field that increasingly integrates psychological, sociological, and cultural perspectives to understand educational emotions as both individual experiences and socially embedded phenomena. The articles in this volume are organized into three thematic blocks that represent major directions in current research:

(1) Methodological and conceptual advances in the study of emotions in education;
(2) Emotional processes shaping learning engagement, motivation, and student development; and
(3) Emotional dynamics within educational communities, including teachers, institutions, and cultural contexts.

## Block 1: methodological and conceptual advances in educational emotion research

The first group of contributions focuses on methodological developments and conceptual frameworks that enable more precise analysis of emotional processes in educational contexts. As research on emotions in education expands globally, the need for culturally sensitive measurement tools and innovative analytical approaches becomes increasingly important. Several studies in this volume address this challenge through psychometric validation and instrument adaptation. Yu and Shi examine the Chinese adaptation of the Emotion Regulation Strategies for Artistic Creative Activities Scale (ERS-ACA-C), demonstrating its reliability and validity among art and design students. Their findings contribute to the growing literature on emotional regulation in creative learning environments, highlighting how emotional management strategies influence artistic performance and creative engagement.

Similarly, Xu et al. investigate the cross-cultural adaptation of the Berkeley Expressivity Questionnaire among Chinese international students studying in Malaysia. Their analysis reveals a four-factor structure that diverges from the original model, underscoring how cultural norms surrounding emotional expression influence the psychometric structure of emotional assessment tools. This study illustrates the importance of adapting psychological measures to account for cultural differences in emotional expression and regulation.

Beyond traditional psychometric approaches, the volume also includes studies employing advanced computational modeling techniques to analyze emotional processes in learning contexts. Hao introduces the ED-CM-MP model, an integrated framework combining dynamic sentiment recognition, cultural adjustment modeling, and temporal prediction to analyze motivation in second language acquisition. By integrating machine learning architectures such as DistilBERT, GraphSAGE, and Temporal Fusion Transformers, the model significantly improves accuracy and efficiency in predicting learning motivation across cross-cultural language learning environments.

Complementing these methodological contributions, Liu and Cho provide a literature review examining the structure of teacher-related boredom in foreign language classrooms. Drawing on Control-Value Theory and previous empirical studies, the authors identify key factors—including teacher emotional support, teacher-student rapport, feedback practices, and teacher burnout—that contribute to boredom in language learning contexts. Their analysis offers a conceptual synthesis that can guide future empirical research on emotions in second language education. Finally, Zhang presents a socio-cognitive linguistic analysis of the Mandarin construction “V+ta+XP,” illustrating how socio-cultural norms and emotional communication interact in language evolution. By integrating construction grammar, sociolinguistic variation, and politeness theory, the study highlights the role of emotional and social pressures in shaping linguistic structures and pragmatic adaptation.

These studies demonstrate the field's growing methodological diversity, combining psychometrics, computational modeling, linguistic analysis, and theoretical synthesis to advance the scientific study of emotions in educational contexts.

## Block 2: emotional processes in learning, motivation, and student development

The second block of articles examines how emotional experiences influence learning engagement, motivation, and academic development across different educational stages. These studies emphasize the dynamic interplay between emotions, motivation, and social support in shaping students' educational trajectories. Several contributions focus on emotional engagement in language learning contexts. Zeng et al. investigate how academic emotions influence engagement in English listening activities among Chinese university students. Their findings reveal that positive emotions such as enjoyment significantly enhance engagement, while negative emotions such as hopelessness and anger reduce behavioral and affective participation. Importantly, the authors demonstrate that teacher autonomy support can mediate these relationships, transforming certain emotions into constructive motivational drivers.

Similarly, Kuzu and Egmir explore socio-emotional readiness in fifth-grade English classrooms in Türkiye. Using a responsive curriculum evaluation framework, the study reveals how emotional safety, peer relationships, and classroom belonging shape students' willingness to participate in communicative language learning activities. The findings highlight the importance of classroom ecology and emotional climate in enabling effective language learning.

Other studies examine emotional processes in earlier educational stages. Wu and Song analyze how affective teacher scaffolding during digital picturebook reading influences preschool children's emotional engagement and social attention. Their results show that emotionally supportive instructional strategies significantly enhance children's positive engagement without reducing cognitive attention to the text, illustrating the pedagogical value of integrating emotional support into early literacy practices.

The emotional development of students is also shaped by family environments. Robledo et al. investigate primary school students' writing attitudes and the role of perceived family support in shaping writing practices. Their findings reveal that family engagement—particularly maternal support—plays a significant role in fostering positive attitudes toward writing, highlighting the importance of home learning environments in literacy development.

Educational experiences also influence broader social and emotional competencies. Rogoski and Pfeiffer-Flores examine the effects of the LuDiCa dialogic reading method on empathy development among children. Through interactive reading sessions centered on stories about immigrants and refugees, the study demonstrates how narrative engagement can promote both interpersonal empathy and literary empathy, fostering deeper understanding of diverse social experiences. Finally, Öncü investigates motivational factors influencing medical career choice among first-year university students using frameworks derived from Social Cognitive Career Theory and Expectancy-Value Theory. The findings indicate that intrinsic motivation, self-efficacy beliefs, and moral commitment play central roles in shaping students' professional aspirations, illustrating how emotional and motivational factors contribute to early professional identity formation.

Studies in this block underscore the central role of emotions in learning processes, demonstrating how emotional experiences influence motivation, engagement, empathy, and academic development across multiple stages of education.

## Block 3: emotional dynamics in educational communities, institutions, and cultural contexts

The final group of articles shifts attention from individual learning processes to the broader educational environments in which emotions are embedded. These studies examine how institutional cultures, professional identities, and social relationships shape emotional experiences among educators and students. Several contributions focus on teacher emotions and professional identity. Li et al. investigate how perceived organizational support influences professional identity among kindergarten teachers in China. Their findings reveal that achievement goal orientations mediate the relationship between institutional support and professional identity, while job stress moderates these effects. The study highlights how organizational contexts shape teachers' emotional experiences and professional development.

Similarly, Jin and Kim explore identity transformation among English teachers working in Chinese private universities. Using narrative inquiry and positioning theory, the authors show how teachers navigate structural constraints and emotional challenges as they construct professional identities. Their findings reveal the importance of recognition, institutional power dynamics, and discursive positioning in shaping teachers' professional trajectories. Teacher emotions are also examined in early career stages. Xiao investigates teaching anxiety among novice Chinese language teachers, identifying evaluation pressure, instructional demands, and classroom management challenges as major sources of anxiety. The study further demonstrates the effectiveness of targeted support interventions in reducing anxiety and turnover intentions among beginning teachers.

Other studies explore emotional dynamics within academic training environments. Xie et al. analyze the relationship between supervisor–group culture, age, and burnout among Chinese doctoral students. Through qualitative interviews, the authors reveal how different supervisory ecologies influence students' stress trajectories, illustrating the role of academic culture in shaping emotional wellbeing and professional development during doctoral training. Institutional and cultural contexts also influence emotional experiences in education. Jin et al. investigate how family cultural capital affects academic achievement through the mediating mechanisms of habitus and field, drawing on Bourdieu's theory of practice. Their results demonstrate how familial resources interact with institutional environments to shape educational outcomes.

Finally, Li and Fan explore the integration of cultural sustainability and student wellbeing through a bilingual Tai Chi course in higher education. Grounded in Self-Determination Theory and embodied cognition, the study shows how embodied practices can support psychological wellbeing while fostering cultural heritage and social sustainability. Taken together, the studies in this block demonstrate how emotions in education are deeply embedded within institutional structures, cultural traditions, and social relationships. They highlight the importance of understanding educational emotions not only as individual psychological processes but also as socially situated experiences shaped by broader cultural and organizational contexts.

The studies compiled in Volume V illustrate the continued expansion of research on emotions in educational contexts. Across diverse disciplinary perspectives and methodological approaches, the contributions demonstrate how emotional experiences influence learning processes, professional identities, and institutional cultures. Three major trends emerge from this collection of research. First, there is a growing emphasis on methodological innovation, including cross-cultural measurement validation and computational modeling techniques capable of capturing complex emotional dynamics. Second, the volume highlights the importance of emotional processes in learning and motivation, demonstrating how emotions shape engagement, empathy, and professional aspirations across educational stages. Third, the studies underscore the significance of institutional and cultural contexts, showing how organizational structures, family environments, and cultural traditions shape emotional experiences in education.

By integrating these perspectives, Volume V advances the understanding of how emotions and culture interact within educational systems. Ultimately, the research presented in this volume contributes to the development of more responsive, inclusive, and emotionally informed educational practices capable of addressing the challenges of contemporary learning environments.

